# Biodegradable iron oxide nanoparticles for intraoperative parathyroid gland imaging in thyroidectomy

**DOI:** 10.1093/pnasnexus/pgac087

**Published:** 2022-06-11

**Authors:** Weihui Zheng, Chun Liu, Jiaoyue Jin, Wei Sun, Jianqiang Zhao, Ming Zhao, Shili Yao, Bing Zhu, Fan Chen, Jinbiao Shang, Kejing Wang, Peng Guo, Jiangjiang Qin, Xiangdong Cheng

**Affiliations:** Key Laboratory of Head and Neck Cancer Translational Research of Zhejiang Province, Zhejiang Cancer Hospital, Hangzhou, Zhejiang 310022, China; The Cancer Hospital of the University of Chinese Academy of Sciences, Zhejiang Cancer Hospital, Hangzhou, Zhejiang 310022, China; Institute of Basic Medicine and Cancer (IBMC), Chinese Academy of Sciences, Hangzhou, Zhejiang 310022, China; Institute of Basic Medicine and Cancer (IBMC), Chinese Academy of Sciences, Hangzhou, Zhejiang 310022, China; The Cancer Hospital of the University of Chinese Academy of Sciences, Zhejiang Cancer Hospital, Hangzhou, Zhejiang 310022, China; The Cancer Hospital of the University of Chinese Academy of Sciences, Zhejiang Cancer Hospital, Hangzhou, Zhejiang 310022, China; Key Laboratory of Head and Neck Cancer Translational Research of Zhejiang Province, Zhejiang Cancer Hospital, Hangzhou, Zhejiang 310022, China; The Cancer Hospital of the University of Chinese Academy of Sciences, Zhejiang Cancer Hospital, Hangzhou, Zhejiang 310022, China; Key Laboratory of Head and Neck Cancer Translational Research of Zhejiang Province, Zhejiang Cancer Hospital, Hangzhou, Zhejiang 310022, China; The Cancer Hospital of the University of Chinese Academy of Sciences, Zhejiang Cancer Hospital, Hangzhou, Zhejiang 310022, China; Institute of Basic Medicine and Cancer (IBMC), Chinese Academy of Sciences, Hangzhou, Zhejiang 310022, China; Institute of Basic Medicine and Cancer (IBMC), Chinese Academy of Sciences, Hangzhou, Zhejiang 310022, China; Key Laboratory of Head and Neck Cancer Translational Research of Zhejiang Province, Zhejiang Cancer Hospital, Hangzhou, Zhejiang 310022, China; Key Laboratory of Head and Neck Cancer Translational Research of Zhejiang Province, Zhejiang Cancer Hospital, Hangzhou, Zhejiang 310022, China; The Cancer Hospital of the University of Chinese Academy of Sciences, Zhejiang Cancer Hospital, Hangzhou, Zhejiang 310022, China; Key Laboratory of Head and Neck Cancer Translational Research of Zhejiang Province, Zhejiang Cancer Hospital, Hangzhou, Zhejiang 310022, China; The Cancer Hospital of the University of Chinese Academy of Sciences, Zhejiang Cancer Hospital, Hangzhou, Zhejiang 310022, China; Institute of Basic Medicine and Cancer (IBMC), Chinese Academy of Sciences, Hangzhou, Zhejiang 310022, China; Key Laboratory of Prevention, Diagnosis and Therapy of Upper Gastrointestinal Cancer of Zhejiang Province, Zhejiang Cancer Hospital, Hangzhou, Zhejiang 310022, China; Institute of Basic Medicine and Cancer (IBMC), Chinese Academy of Sciences, Hangzhou, Zhejiang 310022, China; Key Laboratory of Prevention, Diagnosis and Therapy of Upper Gastrointestinal Cancer of Zhejiang Province, Zhejiang Cancer Hospital, Hangzhou, Zhejiang 310022, China; The Cancer Hospital of the University of Chinese Academy of Sciences, Zhejiang Cancer Hospital, Hangzhou, Zhejiang 310022, China; Key Laboratory of Prevention, Diagnosis and Therapy of Upper Gastrointestinal Cancer of Zhejiang Province, Zhejiang Cancer Hospital, Hangzhou, Zhejiang 310022, China

**Keywords:** iron oxide nanoparticles, parathyroid gland, contrast agent, biodegradability, thyroidectomy

## Abstract

Parathyroid gland (PG) injury is the most common complication of thyroidectomy owing to the lack of approaches for surgeons to effectively distinguish PGs from surrounding thyroid glands (TGs) in the operation room. Herein, we report the development of biodegradable iron oxide nanoparticles (IONPs) as a promising contrast agent candidate for intraoperative PG visualization. We elucidated that locally administrated dark-colored IONPs readily diffuse in TGs but cannot infiltrate tissue-dense PGs, yielding a distinguishable contrast enhancement between PGs and TGs by naked eye observation. We performed unbiased and quantitative in vivo screenings to optimize particle size and concentration of IONPs for PG/TG contrast enhancement. Moreover, in vivo applications of IONPs via the local administration route demonstrate no adverse toxicities and can be biodegraded in the thyroid microenvironment within 3 months. To our knowledge, these promising findings provide the first in vivo evidence that IONPs can serve as a safe, biodegradable, and effective contrast agent candidate for improving PG visualization in thyroidectomy.

Significance Statement Thyroid cancer is the most prevalent endocrine malignancy worldwide and thyroidectomy is a well-established curative modality for treating thyroid cancer. However, parathyroid gland (PG) injury is the most common complication of thyroidectomy owing to the lack of approaches for surgeons to effectively distinguish PGs from surrounding thyroid glands (TGs) in the operation room. In this presented study, we report the development of biodegradable iron oxide nanoparticles (IONPs) as a promising contrast agent candidate for intraoperative PG visualization. We believe that our work merits the field of surgery due to its novelty and potentially high impact in the fields of advanced material-based surgery navigation.

## Introduction

Thyroidectomy is a well-established curative modality for treating thyroid cancer, the most prevalent endocrine malignancy worldwide (1–3). The clinical outcome for thyroidectomy-treated patients achieved a satisfying 5-year survival rate of over 90% (1, 4). Nevertheless, this surgical modality is severely limited by post-thyroidectomy hypocalcemia, an electrolyte derangement condition, as the most common complication of thyroidectomy (5–7). Post-thyroidectomy hypocalcemia is predominantly caused by direct trauma, ischemia, or removal of parathyroid glands (PG) during the operation, owing to the fact that surgeons lack the ability to reliably identify PGs from thyroid glands (TG) and other surrounding tissues (e.g. lymph nodes and peripheral fat particles) in real-time in the operation room, since they have nearly identical anatomy and appearance. For instance, Kovatch et al. (5) reported that 27% of thyroidectomy-treated patients experienced transient hypocalcemia and, unfortunately, approximately 12% of them cannot recover and suffer permanent hypocalcemia conditions requiring lifetime drug medications, highlighting a critical unmet need for developing effective PG protection approaches for thyroidectomy (5).

Contrast agent-enhanced imaging is a viable surgical navigation approach to enhance a surgeon's ability to visually detect occult tumors or native tissues, which has been clinically used for tumor marginal excision and lymph node tracing (8–10). Several conventional small molecule fluorescent dyes (e.g. methylene blue and indocyanine green) and nanomaterials have been approved for these indications (9, 10). Among these contrast agents, carbon nanoparticles (CNPs) have been clinically adopted to negatively stained TGs for PG identification indications for their effectiveness (11, 12). However, carbon nanomaterials including CNPs are well-known for their nonbiodegradability and their human applications have been extensively concerned by their poorly understood genotoxicity and carcinogenic potentials (13–18). Thus, we reasoned that replacing CNPs with a biodegradable contrast agent featuring a better human safety profile will bring substantial benefits to patients receiving thyroidectomy by reducing the potential health hazards of intraoperative PG imaging.

In this study, we addressed this critical challenge by developing biodegradable iron oxide nanoparticles (IONPs) as a safe and effective contrast agent for intraoperative PG imaging in thyroidectomy. IONP is a biodegradable inorganic nanomaterial with outstanding human safety profiles, which has been clinically approved for several human applications (19–22). For instance, intravenous administration of Monofer, an IONP suspension, has been approved by FDA and NMPA for treating iron deficiency anemia (23–25). Taking advantage of its biodegradability and human safety, we hypothesized that IONP may be used as a promising contrast agent candidate for intraoperative PG imaging in thyroidectomy. We validated this hypothesis by quantitatively evaluating the contrast-enhancing capability of IONPs for intraoperative PG imaging, in a head-to-head comparison with clinically used CNPs using rat and rabbit models. Additionally, we performed unbiased and quantitatively in vivo screenings to identify the optimal particle size and concentration of IONPs for intraoperative PG imaging. Our findings strongly support the translational application of IONPs as a novel and promising contrast agent for intraoperative PG imaging in thyroidectomy.

## Results

**Fig. 1.**
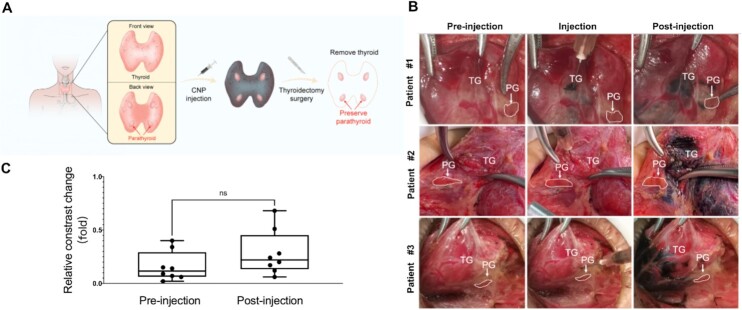
Quantification of the gold clinical standard of intraoperative PG imaging in thyroidectomy. (A) Schematic illustration of CNP-enhanced PG imaging in thyroidectomy surgery. (B) Representative images of CNP-enhanced PG imaging at different time points of pre-injection, injection, and post-injection. (C) Quantified PG/TG contrasts at pre- and post-CNP injection (*n* = 8 per group).

To develop a best-in-class intraoperative PG imaging contrast agent, we first assess the effectiveness of CNPs in clinical settings as the gold clinical standard. In this clinical study, eight patients with thyroid cancer (TNM stage I, *n* = 7) and nodular goiter (*n* = 1) were enrolled (details of patient characteristics were listed in Table S1, Supplementary Material) and all received intraoperative CNP-enhanced PG imaging during their thyroidectomy operation in accordance to Perioperative Period of Parathyroid Gland Function Protection Guide in China (2018 Edition) (26) and the laws and regulations of China, which was illustrated in Figure 1(A). Typically, human TGs present in a butterfly shape in the front of the trachea and four PGs embedded in the back of TGs, which locations can randomly vary based on individual differences and ectopia. Figure 1(B) and Figure S1 (Supplementary Material) show representative images of eight patients receiving intraoperative PG imaging during thyroidectomy operations. When their TGs were surgically exposed, it was obscure for surgeons to precisely identify PG from TG by naked eye observation without CNP enhancing due to their highly similar colors and textures. Next, CNPs were locally administrated into TGs and negatively stained TGs into dark color without staining PGs, which yielded a distinguishable contrast enhancement between PG and TG (PG/TG contrast) by visual speculation in real-time (Figure 1C), allowing surgeons to protect and preserve the PGs in the operation. To measure these clinically meaningful PG/TG contrast changes, we quantified the greyscale value from the region of interest (ROI) in representative photograph pre- and post-CNP injection using Image J software. Our results revealed that CNPs substantially increased PG/TG contrasts by approximately 81% from 0.21 to 0.38 but were not statistically significant (*P* = 0.245). Since this 81% increase of PG/TG contrast is sufficient for surgeons to distinguish PG from their surrounding tissues by naked eye observation, it was selected as a gold clinical standard to guide the development of next-generation PG imaging probes.

**Fig. 2.**
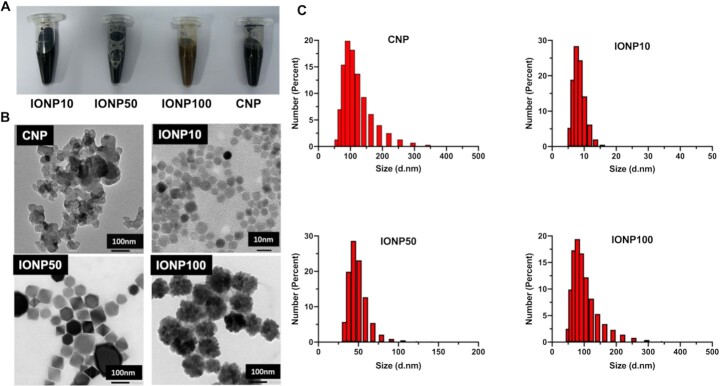
Preparation and characterization of biodegradable IONPs as PG contrast agent candidates. (A) Representative images of IONPs at different particle diameters: 10 nm (IONP10), 50 nm (IONP50), and 100 nm (IONP100). CNPs were used as controls. (B) Representative TEM images of CNP and IONPs. (C) Dynamic light scattering characterization of CNP and IONPs.

IONPs were selected as a potential contrast agent candidate for intraoperative PG imaging because they have highly similar physical properties to CNPs in their color, morphology, and structure, meanwhile owning superior biodegradability and human safety. In order to identify optimal particle size, a panel of IONPs with different diameters including 10 nm (IONP10), 50 nm (IONP50), and 100 nm (IONP100) were characterized for intraoperative PG imaging. All three IONPs exhibit dark brown or black colors that are similar to clinically used CNPs (Figure 2A). Under transmission electron microscopy (TEM) observation (Figure 2B), all three IONPs are better dispersed and more spherical in shape in comparison with CNPs. Their hydrodynamic diameters were determined as 8.32, 48.79, and 97.18 nm with PDIs less than 0.3, featuring their uniformity. In contrast, clinically used CNPs showed a relatively larger hydrodynamic diameter of 114.95 nm (Figure 2B). Their surface charges were characterized as −4.44 ± 0.24 mV for IONP10, −11.97 ± 0.50 mV for IONP50, and −9.62 ± 0.31 mV for IONP100, respectively. These negatively charged IONPs are advantageous in avoiding nonspecific absorption and forming aggregates via electrostatically interacting with negatively charged cells and extracellular matrix proteins in the thyroid microenvironment.

**Fig. 3.**
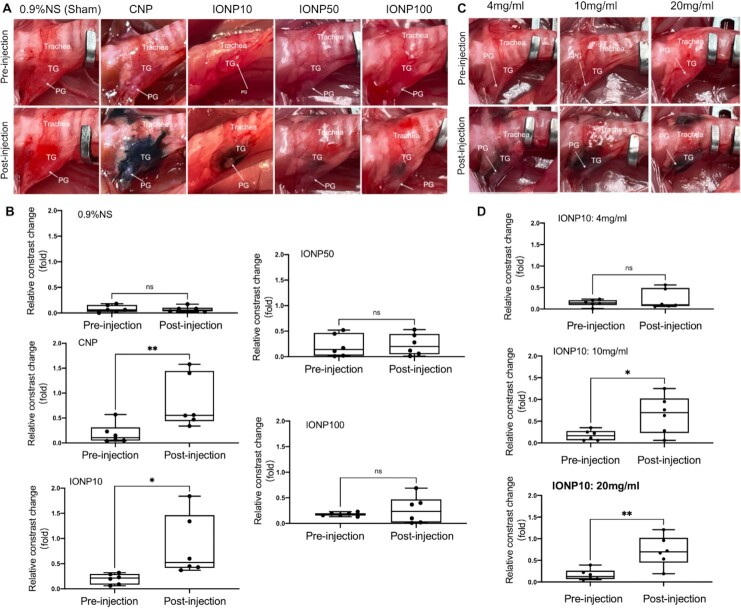
Optimization of IONP particle size and concentration for intraoperative PG imaging. Representative images (A) and quantified PG/TG contrasts (B) of intraoperative PG imaging by using PBS (Sham), CNP, IONP10, IONP50, and IONP100 at pre- and post-injection (*n* = 6 per group). Representative images (C) and quantified PG/TG contrasts (D) of intraoperative PG imaging by using IONP10 at 4, 10, and 20 mg/mL at pre- and post-injection (*n* = 6 per group). NS not significant, * *P* < 0.05, ** *P* < 0.01, and *** *P* < 0.001.

We next performed an unbiased and quantitative in vivo screening to identify the optimal IONP size for intraoperative PG imaging using a rat model. Sex and age-matched SD rats were divided into five groups (*n* = 6 per group) and their TGs were surgically exposed and received a local injection of PBS, CNP, IONP10, IONP50, or IONP100 at an equivalent particle concentration of 10 mg/mL. Representative images of intraoperative PG imaging at pre- and post-PBS, CNP, or IONP injection were shown in Figure 3(A) and Figure S2 (Supplementary Material). IONP10 exhibits the highest contrast enhancement between PGs and TGs, substantially outperforming IONP50 and IONP100. The PG/TG contrast of IONP10 was quantitatively determined as 0.199 and 0.839 at pre- and postinjection (Figure 3B). In contrast, clinically used CNPs demonstrated a darker color with a higher tissue infiltration than IONP10s, which errantly infiltrated the extent of PGs and normal trachea tissues (Figure 3A). CNPs mediates an increase of PG/TG contrast from 0.184 to 0.817, at an equivalent level with IONP10s (Figure 3B).

**Fig. 4.**
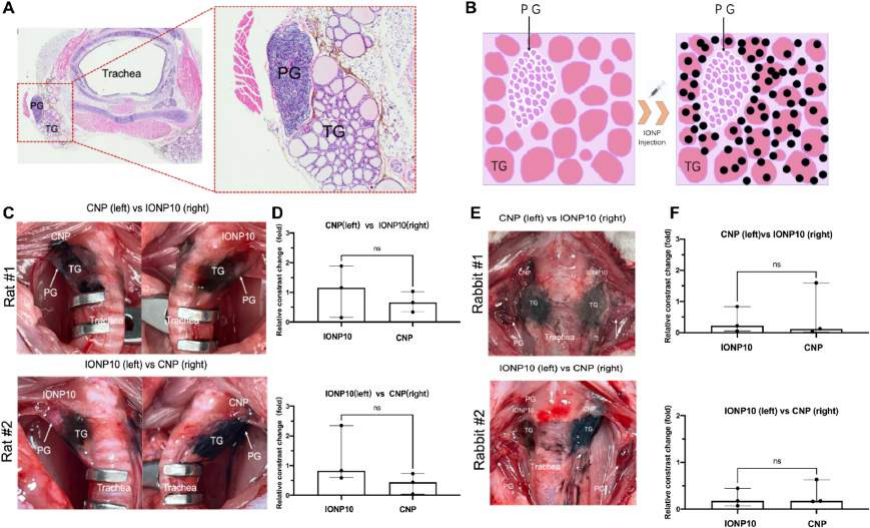
Head-to-head comparison of IONP10 and CNP for intraoperative PG imaging. (A) Representative histology images of isolated rat trachea, TG, and PG post-IONP10 treatment. (B) Schematic illustration of locally administration of IONP10 in thyroid microenvironment for PG visualization. Representative images (C) and quantified PG/TG contrasts (D) of IONP10 and CNP locally injected in symmetrical TGs of the same rat. Representative images (E) and quantified PG/TG contrasts (F) of IONP10 and CNP locally injected in symmetrical TGs of the same rabbit. (*n* = 3 per group). NS not significant, * *P* < 0.05, ** *P* < 0.01, and *** *P* < 0.001.

We elucidated the underlying mechanism of IONP10-enhanced PG imaging by performing a histology study. The PG, TG, and trachea tissues from IONP10-treated rats were isolated for H&E staining. Their cross-section images reveal that TG tissues are enriched with many loose lumens, demonstrating an obvious lower tissue density than PG (Figure 4A). In comparison, PG features a denser tissue that is composed of closely aligned chief cells and eosinophils. This histological finding supports our clinical and animal observations that IONP10s or CNPs can readily infiltrate TGs but not PGs (Figure 4B). The imbalanced nanoparticle distribution between PG and TG yields a distinguishable contrast for visual speculation in real-time. Based on these promising findings, IONP10 was selected as the optimal particle size for intraoperative PG imaging. We next optimized different IONP particle concentrations for PG imaging. IONP10s with escalating particle concentrations (4, 10, and 20 mg/mL) were locally administrated into rat TGs, giving rise to increasing PG/TG contrasts as 0.225, 0.656, and 0.712, respectively (Figure 3C and D; Figure S3, Supplementary Material). Since IONP10 at 20 mg/mL yields the highest PG/TG contrast increase of 434%, it was selected as the optimal IONP10 concentration for intraoperative PG imaging.

To determine whether IONP10 can reliably replace clinically used CNP in intraoperative PG imaging, we performed a head-to-head comparison of their effectiveness in the same animal. IONP10 and CNP were separately injected into symmetrical TGs of the same rat (*n* = 3 per group) at equivalent particle dose and concentration (20 mg/mL). Both IONPs and CNPs readily dispersed in TGs and yield a distinguishable contrast difference between TGs and PGs (Figure 4C; Figure S4, Supplementary Material). Quantitative analyses in Figure 4(D) revealed that IONP10s demonstrated a 1.75-fold higher PG/TG contrast (1.16) than that of CNP (0.66). This head-to-head comparison was repeated by switching the injected TG sides of IONP10 and CNP, and once again, IONP10 demonstrated a 1.88-fold higher PG/TG contrast (0.83) than that of CNP (0.44) regardless of TG sidedness (Figure 4C and D). We validated these important findings by performing the same head-to-head comparison of IONP10 and CNP in a rabbit model, which bodyweight is approximately 10-fold heavier than an SD rat (Figure 4E and F). IONP10 and CNP were separately injected into symmetrical TGs of the same rabbit (*n* = 3) under identical conditions. In consistence with our findings obtained from the rat model, optimal IONP10 yields a slightly higher or equivalent PG/TG contrast in comparison with CNPs regardless of TG sidedness of the same rabbit (*n* = 3 per group). These in vivo results proved that IONP10 is an effective contrast agent for intraoperative PG imaging with equivalent or superior efficacy as clinically used CNPs.

**Fig. 5.**
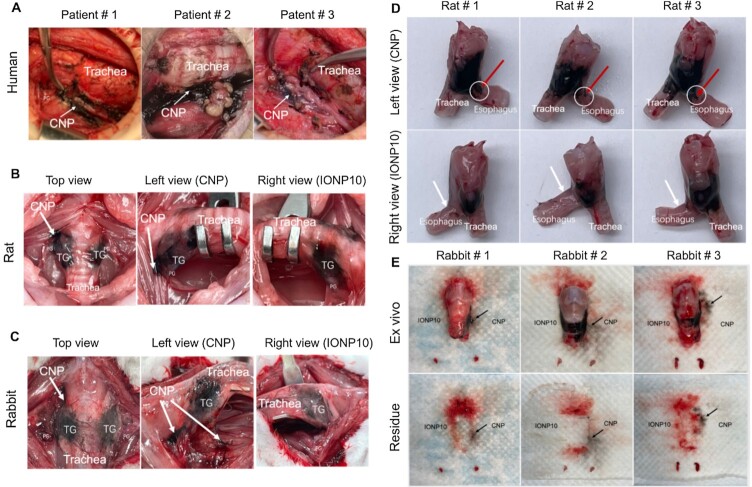
Head-to-head comparison of IONP10 and CNP for operation site contamination. (A) Representative images of operation site contamination by CNPs at post-thyroidectomy in patients (*n* = 3 per group). Representative images of operation site contaminations by CNP and IONP10 in rat (B) and rabbit (C) models. Representative ex vivo images of operation site contaminations by CNP and IONP10 in rat (D) and rabbit (E) models.

Operation site contamination is an unneglectable drawback of clinically used CNPs that commonly happens during intraoperative PG imaging procedures (27). Representative images of post-thyroidectomy operation sites in patients (Figure 5A) revealed the infiltrative patterns of locally administrated CNPs after TG resection. We found that many CNPs have already infiltrated TG surrounding tissues including normal trachea and recurrent laryngeal nerves of these patients, which cannot be cleaned and may permanently reside within these normal tissues due to the nonbiodegradability of CNPs, giving rise to worrisome safety concerns about the human application of CNPs. To determine whether IONP10 can avoid this complication, we compared the operation site contamination of IONP10 and CNP in a rat model (Figure 5B; Figure S4, Supplementary Material). IONP10 and CNP were separately injected into the symmetric PGs of the same rat (*n* = 3 per group) under the same conditions, and CNP rapidly infiltrated peripheral anterior cervical muscles surrounding TGs in vivo as expected. In comparison, optimized IONP10s only diffuse within PGs without contaminating any surrounding tissues in operation sites. Ex vivo tissue images further confirmed that CNPs (left TG) obviously contaminated the normal esophagus during intraoperative PG imaging, whereas IONP10s (right TG) were almost localized within the TG without infiltrating the esophagus (Figure 5D). These findings were further validated in a rabbit model. Similarly, CNPs rapidly diffused out from the injected TG and infiltrated surrounding peripheral anterior cervical muscles (Figure 5C). Such contaminations were not observed in the IONP10-injected side of the same rabbit. Ex vivo images (Figure 5D; Figure S5, Supplementary Material) showed that CNPs readily leaked out of injected TGs and contaminated the background substrate within 2 h, whereas IONP10s maintained their localization within injected TGs. To clarify the reason why clinically used CNPs showed higher infiltration activities at even larger sizes in comparison with IONP10s, we performed a thin layer chromatography assay (Figure S6, Supplementary Material) and found that clinically used CNPs contain an excipient named povidone K30, which is an effective lubricant that tremendously enhances the infiltration activity of CNPs. When povidone K30 were removed from the CNP suspension by PBS washing, their high infiltration activity was lost. Meanwhile, we also compared optical colors of CNPs with IONP50s and IONP100s at serially diluted particle concentrations from 0.5 to 20 mg/mL and found that clinically used CNPs are optically darker than tested IONPs at the same concentration (Figure S7, Supplementary Material).

**Fig. 6.**
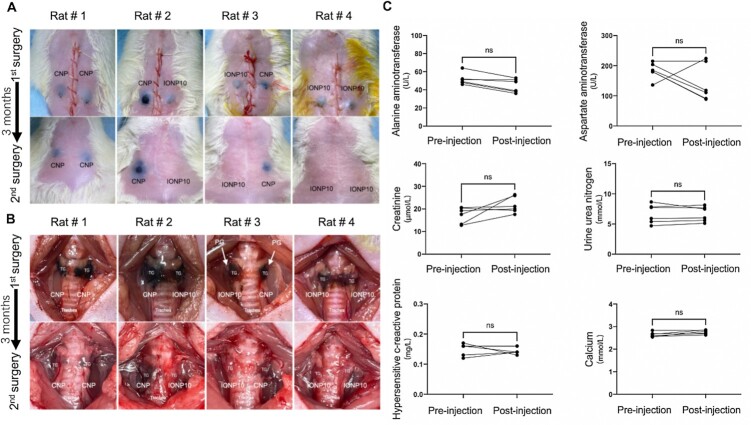
Long-term biodegradation and acute toxicity of IONP10. Representative images of skin observation (A) and operation site observation (B) of CNP and IONP10 biodegradation conditions in rat TG microenvironments for a period of 90 days. (C) Quantified levels of blood biomarkers including ALT, AST, creatinine, urine urea nitrogen, hypersensitive c-reactive protein, and calcium at pre- and post-IONP10 (*n* = 6 per group). NS not significant, * *P* < 0.05, ** *P* < 0.01, and *** *P* < 0.001.

Biodegradability is an incomparable advantage of IONP10 over clinically used CNP for intraoperative PG imaging. To determine whether IONP10 can be degraded in the thyroid microenvironment, we performed a long-term biodegradation study of IONP10s and CNPs in intraoperative PG imaging settings (Figure 6A). IONP10s and CNPs were locally injected into rat TGs under the same conditions. After recovering from the surgery, these rats were further housed for 90 days. Strikingly, after 90 days, most IONP10s were biodegraded and are no longer visible by eye in comparison with nonbiodegradable CNPs. We further surgically exposed IONP10 and CNP-injected TGs to examine their degradation rates. In consistence with the skin observation, most IONP10s were biodegraded in TGs whereas, on the other hand, nonbiodegradable CNPs not only retained in injected TGs but also contaminated surrounding normal tissues of the operation site (Figure 6B). Additionally, we evaluated the acute toxicity of IONP10 for intraoperative PG imaging. Optimal IONP10 were injected into surgically exposed TGs and then the incision was sutured for recovery. Blood samples (*n* = 6 per group) were collected at pre- and 24 h post-IONP10 injection from the same animals and a panel of serum biomarkers was quantitatively determined using blood chemistry (Figure 6C; Figure S8, Supplementary Material). Importantly, two liver toxicity biomarkers (aspartate aminotransferase (AST) and alanine aminotransferase (ALT)) and two renal toxicity biomarkers (Creatinine and urine urea nitrogen) show no obvious changes after IONP injection, indicating IONP10s do not affect normal liver and kidney functions. Similarly, serum calcium and C-reactive protein levels were also unaffected, indicating that IONP10 neither affects the biological functions of TGs and PGs nor induces immune responses. These results strongly support that locally administrated biodegradable IONP10 in TGs is a safe and feasible approach for intraoperative PG visualization.

## Discussion

PG protection and preservation are essential challenges in thyroidectomy. To our knowledge, our study provides the first experimental evidence that biodegradable IONP10s can be used as a safe and effective contrast agent for intraoperative PG visualization, owing to three major advantages: (1) locally administration of IONP10s in TG readily differentiates PGs from surrounding tissues by naked eye observation without requiring external microscopes; (2) IONP10s are composed of iron oxide, a biodegradable and clinically safe inorganic material that is more suitable for human applications than nonbiodegradable CNPs; (3) particle size and concentration of IONP10 are optimized for intraoperative PG imaging, considerably reducing operation site contaminations in comparison with clinically used CNPs.

To date, CNPs represent the most commonly used contrast agents for PG localization and preservation in thyroidectomy as recommended by Perioperative Period of Parathyroid Gland Function Protection Guide in China (2018 Edition) (26). However, the human application of CNPs as an imaging contrast agent has been severely limited by their nonbiodegradability and potential carcinogenic concerns (13–18). In our study, we revealed that CNP cannot be biodegraded within thyroid microenvironments after 90 days (Figure 6) and errantly infiltrated several critical organs surrounding TGs including recurrent laryngeal nerve, trachea, and esophagus in operation sites during intraoperative PG imaging (Figure 5). Notably, recent reports found that carbon nanomaterials have high genotoxicity and carcinogenic potential by inducing DNA damages and interfering gene replication and transcription in the cell nucleus, frequently inducing tumorigenesis in animal models (17, 18). These worrisome findings bring serious long-term safety concerns about human applications of carbon nanomaterials including CNPs. Several alternative technologies have been developed for intraoperative PG imaging applications including near-infrared autofluorescence imaging (28) and Raman spectroscopy (29) for replacing CNPs in clinical practice. There remains a critical unmet need to improve biodegradability and safety profiles of current contrast agents used in intraoperative PG imaging.

Our presented studies showed that IONP is an excellent contrast agent candidate for intraoperative PG imaging. The biodegradation mechanism of IONPs has previously been elucidated that cellular internalized IONPs are metabolized into Fe^3+^ ions in the acidic and enzyme-rich environment of lysosomes, where ferritin and transferrin proteins bind Fe^3+^ ions and subsequently facilitate systematic iron absorption and recycle for many important biological processes including erythropoiesis, liver and heart functions (30–32). Owing to their outstanding human safety and biodegradability, intravenous administration of IONPs has already been clinically approved by FDA and NMPA for treating acute iron deficiency anemia (23–25). Our in vivo results further validated that most IONP10s can be biodegraded within the thyroid microenvironment and are not detectable by eye after 90 days in a head-to-head comparison with CNPs (Figure 6). Moreover, IONPs also demonstrate highly similar physical properties (e.g. color, shape, and morphology) as the clinically used CNPs and the particle size and concentration of IONP10s were optimized for intraoperative PG imaging through unbiased and quantitative in vivo screenings (Figures 3 and 4). The optimized IONP10 demonstrated a 431% and 179% increased PG/TG contrasts in rat and rabbit models, over 2.2-fold higher than the gold clinical standard by CNPs (81%). To our knowledge, these promising results represent the first in vivo demonstration that IONPs can serve as a safe and effective contrast agent for intraoperative PG imaging.

In summary, we have developed and evaluated biodegradable ION10s as a safe and effective contrast agent candidate for intraoperative PG imaging in thyroidectomy. Our study shows that IONP10 has incomparable advantages over clinically used CNPs in human safety profiles, representing a promising translational opportunity for next-generation intraoperative PG imaging contrast agents.

## Materials and Methods

### Materials

The clinically used CNPs were purchased from Chongqing Laimei Pharmaceuticals (Chongqing, China). The IONP10, IONP50, and IONP100 were obtained from the Zhongke Leiming Technology (Beijing, China). All IONPs used in this study were coated with dimercaptosuccinic acid (DMSA), an FDA approved agent for the treatment of lead intoxication (33). Hematoxylin and Eosin-Y were purchased from Thermo Fisher (Waltham, USA). Isoflurane and potassium chloride were purchased from Sigma Aldrich (St Louis, USA).

### Clinical intraoperative PG imaging study

A total of eight patients were enrolled from the Cancer Hospital of the Chinese Academy of Sciences (Zhejiang Cancer Hospital) in China between July 2021 and August 2021. Patients who underwent thyroidectomy for malignant tumors or benign goiter were considered eligible for this study. The procedure of CNP-enhanced PG imaging was performed in accordance with the Perioperative Period of Parathyroid Gland Function Protection Guide in China (2018 Edition) (26) and the laws and regulations of China. All participants provided written informed consent. Standard thyroidectomy procedures were performed to expose thyroid and parathyroid glands. A total of 5mg CNPs was slowly injected into exposed thyroids and PGs were identified through naked eye observation. Representative images were taken at different time points between pre- and post-CNP injection. Intraoperative PG imaging analysis was performed by using Image J software that was used to quantify the greyscale values of TG and PG at pre- and post-CNP injections. The ROIs were drawn around TG and PG from photographs and their relative contrast changes were calculated by comparing the difference of greyscale values between TG and PG at pre- and post-CNP injection.

### Characterization of IONP and CNP

IONPs with three different diameters (10, 50, and 100 nm) were used in this study. The nanoparticulate size and structure of IONP and CNP were characterized by a TEM (JEOL JEM-2100plus). A volume of 50 µg/mL IONP in PBS solution were added to copper grid dropwise and dried for 30 min. Then dried IONP samples were imaged using a TEM at a voltage of 80 kV. The hydrodynamic diameter and zeta potential of IONPs were determined by using a Zetasizer Nano ZS (Malvern, zen3600) at a sample concentration of 50 µg/mL in PBS solution.

### Intraoperative PG imaging in a rat model

Animal studies were performed according to the protocols approved by the Institutional Animal Care and Use Committees of the Institute of Basic Medicine and Cancer, Chinese Academy of Sciences. For intraoperative PG imaging, 6-week-old SD rats (male/female ratio 1/1) were anesthetized with isoflurane and received a longitudinal incision on the neck to surgically expose their TGs, followed by local injection of contrast agents into their TGs using a 30G insulin syringe. For nanoparticle size optimization study, sex and age-matched SD rats were randomly divided into five groups (*n* = 6 per group) and received treatment of 10 µL PBS (sham), CNP, IONP10, IONP50, and IONP100 at an equivalent particle concentration of 10 mg/mL, respectively. Rat TGs and PGs were imaged using a camera at a fixed distance of 20 cm pre- and post-injection. Then rats were euthanized,after overdose of anesthesia,using intracardiac injection of potassium chloride solution. Postmortem, PG, TG, laryngopharynx, and trachea were isolated for ex vivo imaging and PG/TG contrast changes were quantified using the same method as previously described in the clinical intraoperative PG imaging study.

For nanoparticle concentration optimization study, sex and age-matched rats were randomly divided into three groups (*n* = 6 per group) and received local administration of 10 µL IONP10 solutions at 4, 10, and 20 mg/mL in their TGs, respectively. All other parameters and data analysis remain consistent with the previously described intraoperative PG imaging study.

For head-to-head comparison of CNP and IONP10 study, sex and age-matched rats were randomly divided into two groups (*n* = 3 per group) and received local administration of 10 µL IONP10 and CNP solutions at an equivalent concentration of 20 mg/mL in their symmetrical TGs of the same rat. The sidedness of TGs injected with CNP or IONP10 were switched between two treatment groups. All other parameters and data analysis remain consistent with the previously described intraoperative PG imaging study.

### Long-term biodegradability study

Rats, 6-week-old SD (male/female ratio 1/1), were anesthetized and their TGs were surgically exposed and received local administration of IONP10 or CNP at an equivalent dose of 10 µL of 20 mg/mL suspension. Rat PG and TG were imaged at pre- and post-injection. Then the incision on the rat neck was sealed by sutures and the recovered rats were housed for 90 days for biodegradation of CNP and IONP10. After 3 months, the rats previously received IONP10 or CNP injections received second surgery to examine the biodegradation conditions of CNP and IONP10 injected in their TG by imaging.

### Acute toxicity study

Rats, 6-week-old SD (male/female ratio 1/1), were anesthetized and their TGs were surgically exposed and received local administration of IONP10 at an optimized dose of 10 µL of 20 mg/mL suspension. After IONP10-injection, the incision on the rat neck was sealed by sutures and the animals were allowed to recover from surgery for 24 h. Rat blood samples were collected at pre- and post-IONP injection and a panel of blood biomarkers was measured using an automatic biochemical analyzer (Hitachi 7600, Hitachi medical diagnostics) to determine the acute toxicity of IONP10.

### Intraoperative PG imaging in a rabbit model

For the rabbit study, 3-month-old New Zealand rabbits were used for the intraoperative PG imaging (male, *n* = 6 per group). Rabbits were anesthetized by isoflurane and received a longitudinal incision on the neck to surgically expose their TGs, followed by local administration of 10 µL IONP10 or CNP suspension into symmetric TGs of the same rabbit at an equivalent concentration of 20 mg/mL using a 30G insulin syringe. Rabbit TGs and PGs were imaged using a camera pre- and postinjection. Then treated rabbits were euthanized,after overdose of anesthesia,using Intravenous injection of potassium chloride solution. Postmortem, PG, TG, laryngopharynx, and trachea were isolated for ex vivo imaging and PG/TG contrast changes were quantified using the same method as previously described in the rat study.

### Statistical analysis

All of the experimental data were obtained at least in triplicate and are presented as mean ± SD unless otherwise mentioned. Statistical comparison by analysis of variance was performed at a significance level of *P* < 0.05 based on unpaired Student's t tests.

## Supplementary Material

pgac087_Supplemental_Files

## Data Availability

All data is included in the manuscript and/or supporting information.
