# Correction to: Biodegradable iron oxide nanoparticles for intraoperative parathyroid gland imaging in thyroidectomy

**DOI:** 10.1093/pnasnexus/pgad462

**Published:** 2024-01-30

This is a correction to: Weihui Zheng, Chun Liu, Jiaoyue Jin, Wei Sun, Jianqiang Zhao, Ming Zhao, Shili Yao, Bing Zhu, Fan Chen, Jinbiao Shang, Kejing Wang, Peng Guo, Jiangjiang Qin, Xiangdong Cheng, Biodegradable iron oxide nanoparticles for intraoperative parathyroid gland imaging in thyroidectomy, *PNAS Nexus*, Volume 1, Issue 3, July 2022, pgac087, https://doi.org/10.1093/pnasnexus/pgac087

During a retroactive audit conducted by *PNAS Nexus*, it was discovered that this paper was missing a statement acknowledging compliance with the *PNAS Nexus* Human and Animal Participants and Clinical Trials policy:

The non interventional clinical intraoperative PG imaging study was approved by the Medical Ethics Committee of Zhejiang Cancer Hospital.

This error has been corrected in the original article.

