# Supplementary material for: Biodegradable iron oxide nanoparticles for intraoperative parathyroid gland imaging in thyroidectomy
Source: PNAS Nexus. 2022 Jun 11;1(3):pgac087. doi: 10.1093/pnasnexus/pgac087 (PMC9896913; doi:10.1093/pnasnexus/pgac087)
Supplement: pgac087_Supplemental_Files [file pgac087_supplemental_files.zip › PNASNEXUS-PNASNEXUS-2022-00132-s02.docx]

**Table S1.** The clinical and pathological characteristics of 8 patients who received CNP-enhanced intraoperative PG imaging.

|  | Age | Gender | Body Mass Index（BMI, kg/m^2^） | Surgery Type | Diagnosis | TNM Stage |
| --- | --- | --- | --- | --- | --- | --- |
| 1 | 52 | F | 22.86 | Hemithyroidectomy | Thyroid cancer | T1N0M0 |
| 2 | 29 | F | 18.10 | Hemithyroidectomy | Thyroid cancer | T1N1aM0 |
| 3 | 33 | F | 23.74 | Hemithyroidectomy | Thyroid cancer | T1N1aM0 |
| 4 | 58 | M | 20.81 | Hemithyroidectomy | Thyroid cancer | T1N1aM0 |
| 5 | 32 | F | 23.50 | Total thyroidectomy | Thyroid cancer | T1N1aM0 |
| 6 | 64 | F | 21.64 | Hemithyroidectomy | Goiter | None |
| 7 | 48 | F | 21.63 | Total thyroidectomy | Thyroid cancer | T1N0M0 |
| 8 | 48 | F | 23.43 | Hemithyroidectomy | Thyroid cancer | T1N0M0 |
