# Supplementary material for: Biodegradable iron oxide nanoparticles for intraoperative parathyroid gland imaging in thyroidectomy
Source: PNAS Nexus. 2022 Jun 11;1(3):pgac087. doi: 10.1093/pnasnexus/pgac087 (PMC9896913; doi:10.1093/pnasnexus/pgac087)
Supplement: pgac087_Supplemental_Files [file pgac087_supplemental_files.zip › PNASNEXUS-PNASNEXUS-2022-00132-s06.pdf]

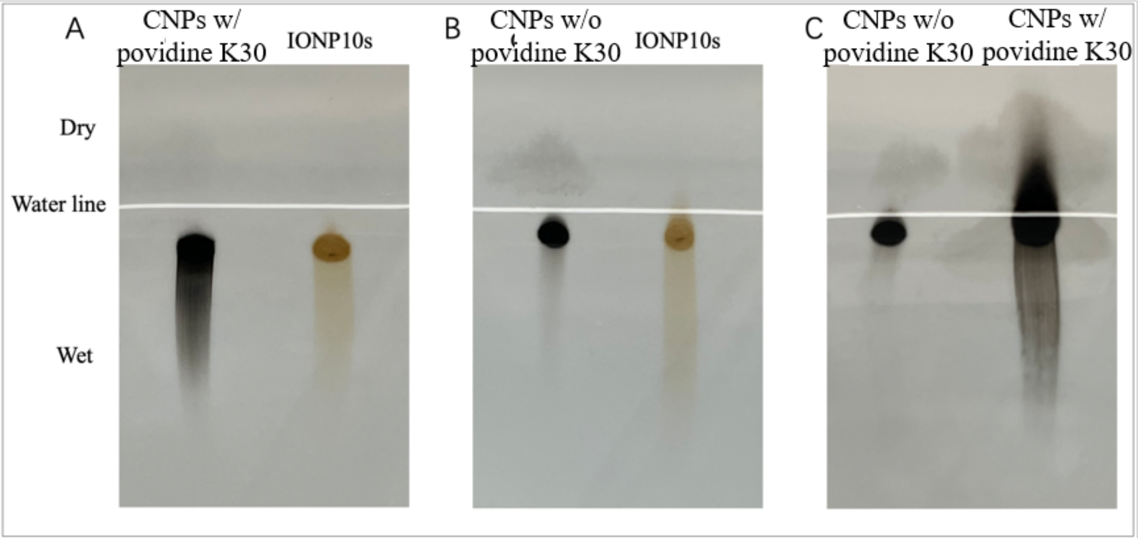

A
CNPs w/ povidine K30
IONP10s
Dry
Water line
Wet
B
CNPs w/o povidine K30
IONP10s
C
CNPs w/o povidine K30
CNPs w/ povidine K30
