# Supplementary material for: Biodegradable iron oxide nanoparticles for intraoperative parathyroid gland imaging in thyroidectomy
Source: PNAS Nexus. 2022 Jun 11;1(3):pgac087. doi: 10.1093/pnasnexus/pgac087 (PMC9896913; doi:10.1093/pnasnexus/pgac087)
Supplement: pgac087_Supplemental_Files [file pgac087_supplemental_files.zip › PNASNEXUS-PNASNEXUS-2022-00132-s09.pdf]

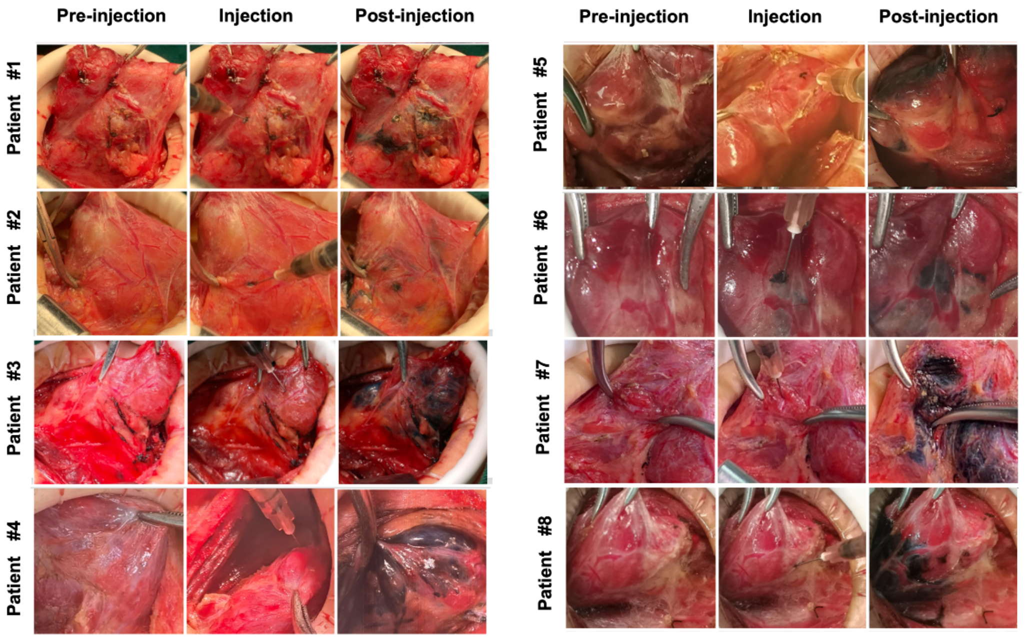
Pre-injection
Injection
Post-injection
Patient #1
Patient #2
Patient #3
Patient #4
Pre-injection
Injection
Post-injection
Patient #5
Patient #6
Patient #7
Patient #8
