# Supplementary material for: Biodegradable iron oxide nanoparticles for intraoperative parathyroid gland imaging in thyroidectomy
Source: PNAS Nexus. 2022 Jun 11;1(3):pgac087. doi: 10.1093/pnasnexus/pgac087 (PMC9896913; doi:10.1093/pnasnexus/pgac087)
Supplement: pgac087_Supplemental_Files [file pgac087_supplemental_files.zip › PNASNEXUS-PNASNEXUS-2022-00132-s01.docx]

**Figures captions**

**Figure S1.** In situ images of 8 patients received CNP-enhanced intraoperative PG imaging at serial time points of pre-injection, injection, and post-injection during thyroidectomy (n=8 per group).

**Figure S2.** In situ images of 6 rats received intraoperative PG imaging by using PBS (Sham), CNP, IONP10, IONP50, and IONP100 at post-injection (n=6 per group).

**Figure S3.** In situ images of 6 rats received intraoperative PG imaging by using 4mg/ml,10mg/ml,20mg/ml of IONP10 at post-injection (n=6 per group).

**Figure S4.** In situ images of intraoperative PG imaging enhanced by CNP and IONP10 injected in symmetrical TGs of the same rat. (n=6 per group).

**Figure S5.** In situ images of intraoperative PG imaging enhanced by CNP and IONP10 injected in symmetrical TGs of the same rabbit. (n=6 per group).

**Figure S6.** The infiltration activity of CNPs with or without povidone K30 in comparison with IONP10s. (A) comparison between CNPs with povidone K30 and IONP10s; (B) comparison between CNPs without povidone K30 and IONP10s; (C) comparison between CNPs with and without povidone K30.

**Figure S7**. The optical color and infiltration activity of CNPs, IONP50s and IONP100s. (A) The optical color of CNPs (with povidone K30), IONP50s and IONP100s at serially diluted particle concentrations. (B) The infiltration activity of CNPs (with povidone K30), IONP50s and IONP100s.

**Figure S8.** A panel of blood biomarker changes at pre-and-post-IONP injection, including cholinesterase, total bile acid, uric acid, total cholesterol, triglyceride, kalium, creatine kinase, albumin, transferrin. (n=6 per group). NS not significant, *P<0.05, ** P<0.01, *** P<0.001.
