# Supplementary figures and images for: Biodegradable iron oxide nanoparticles for intraoperative parathyroid gland imaging in thyroidectomy
Source: PNAS Nexus. 2022 Jun 11;1(3):pgac087. doi: 10.1093/pnasnexus/pgac087 (PMC9896913; doi:10.1093/pnasnexus/pgac087)

Rat #1

Rat #2

Rat #3

Rat #4

Rat #5

Rat #6

4mg/ml

10mg/ml

20mg/ml

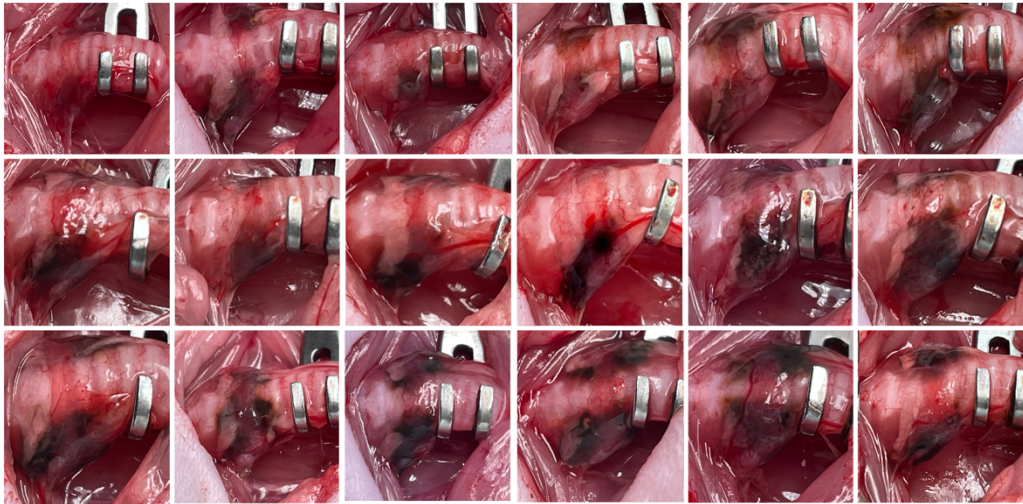

Supplement: pgac087_Supplemental_Files [file pgac087_supplemental_files.zip › PNASNEXUS-PNASNEXUS-2022-00132-s03.pdf]

Left view (CNP)

Right view (IONP10)

Left view (IONP10)

Right view (CNP)

Rat #1

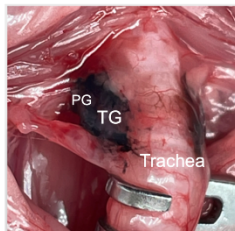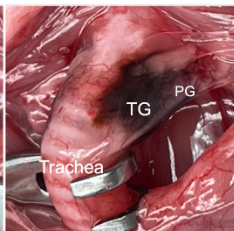

Rat #2

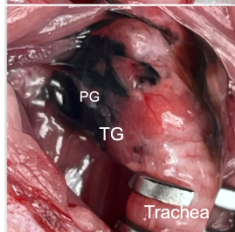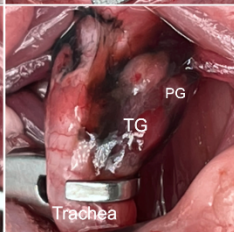

Rat #3

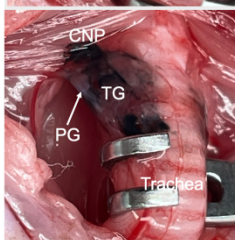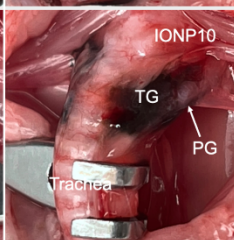

Rat #4

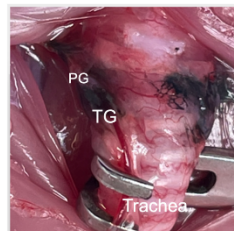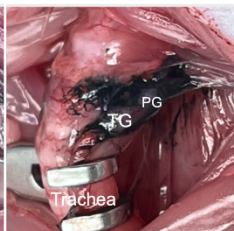

Rat #5

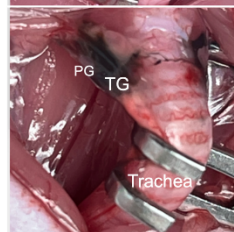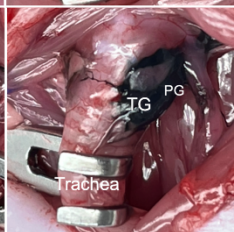

Rat #6

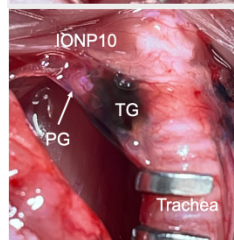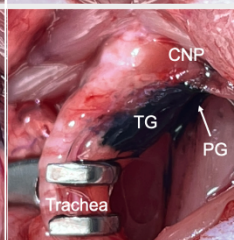

Supplement: pgac087_Supplemental_Files [file pgac087_supplemental_files.zip › PNASNEXUS-PNASNEXUS-2022-00132-s04.pdf]

## CNP (left) vs IONP10 (right)

## CNP (left) vs IONP10 (right)

Rabbit # 1

Rabbit # 2

Rabbit # 3

Rabbit # 4

Rabbit # 5

Rabbit # 6

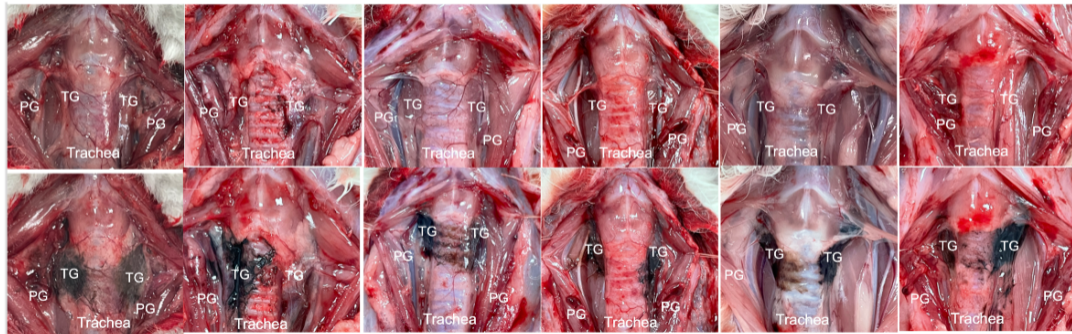

Supplement: pgac087_Supplemental_Files [file pgac087_supplemental_files.zip › PNASNEXUS-PNASNEXUS-2022-00132-s05.pdf]

**A**

20

10

5

1

0.5(mg/ml)

CNPs

IONP50s

IONP100s

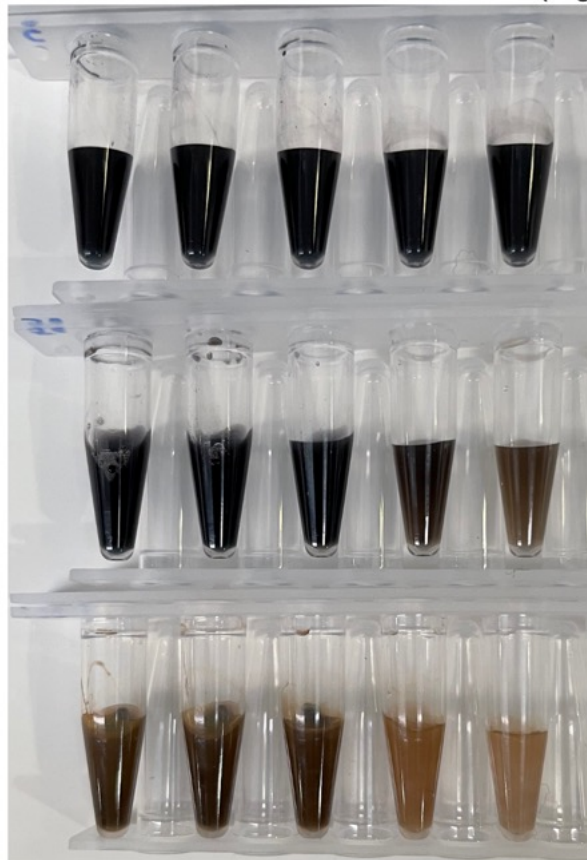**B**

20mg/ml

CNPs

IONP50s

IONP100s

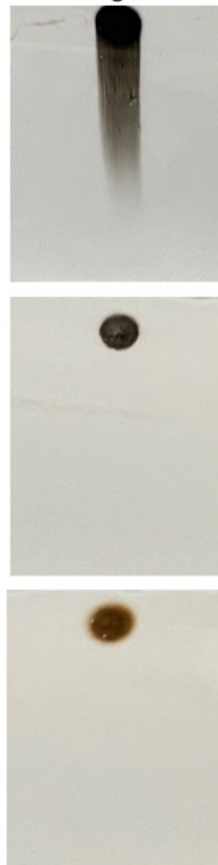

Supplement: pgac087_Supplemental_Files [file pgac087_supplemental_files.zip › PNASNEXUS-PNASNEXUS-2022-00132-s07.pdf]

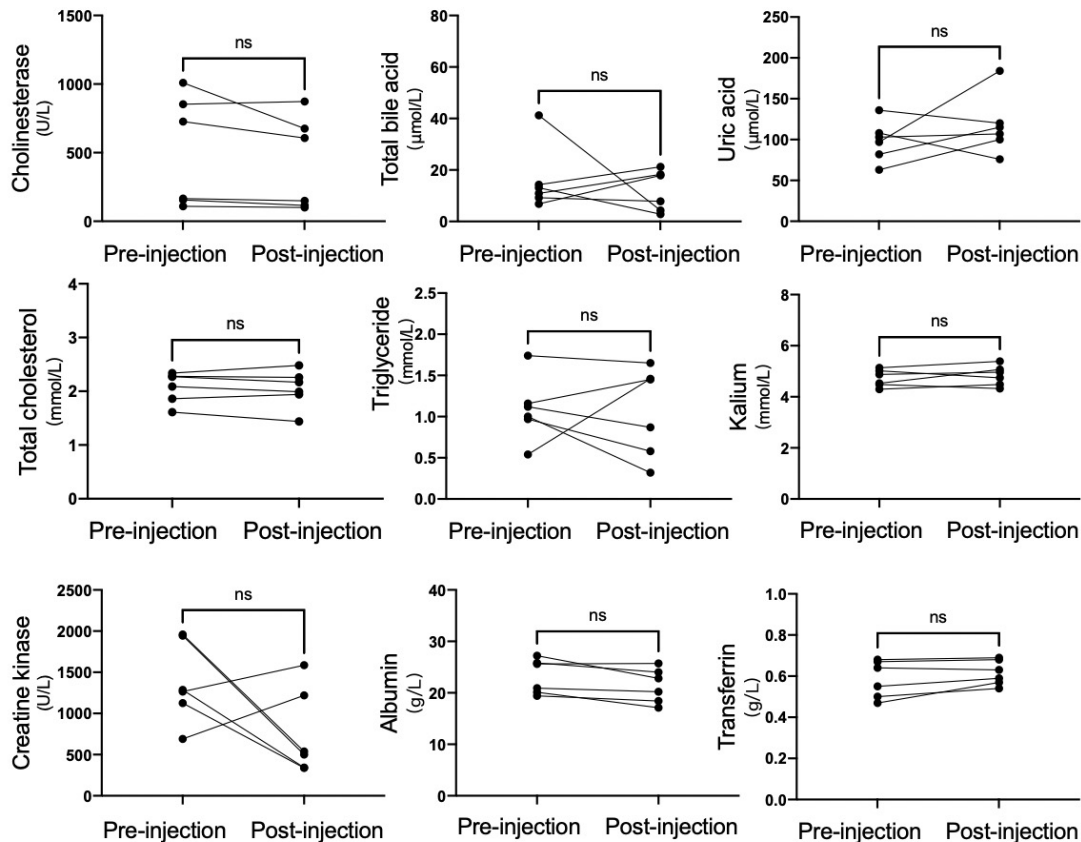

Supplement: pgac087_Supplemental_Files [file pgac087_supplemental_files.zip › PNASNEXUS-PNASNEXUS-2022-00132-s08.pdf]

Rat #1

Rat #2

Rat #3

Rat #4

Rat #5

Rat #6

0.9%NS

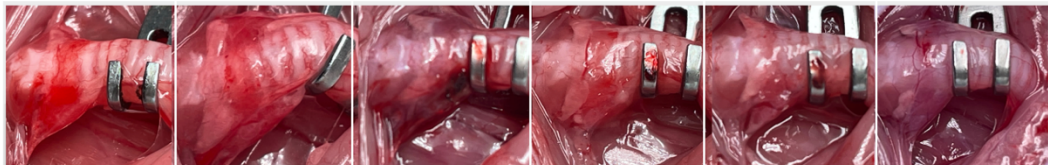

CNP

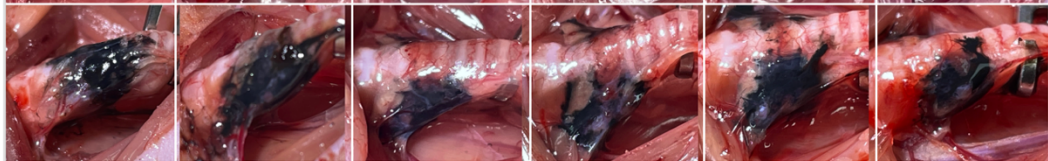

IONP10

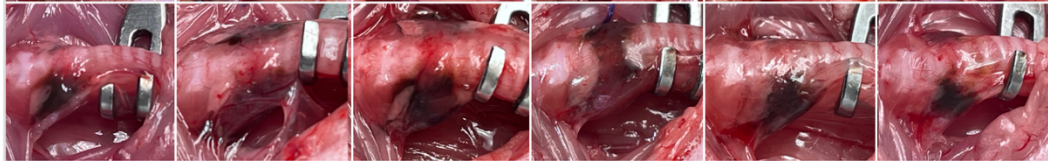

IONP50

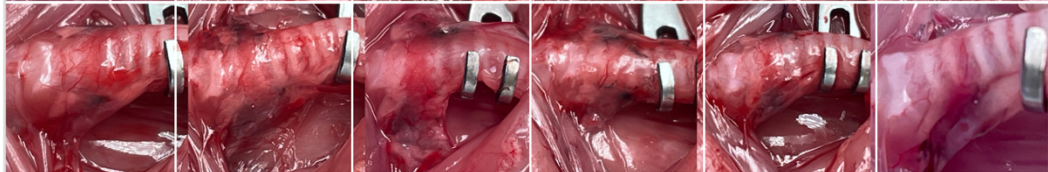

IONP100

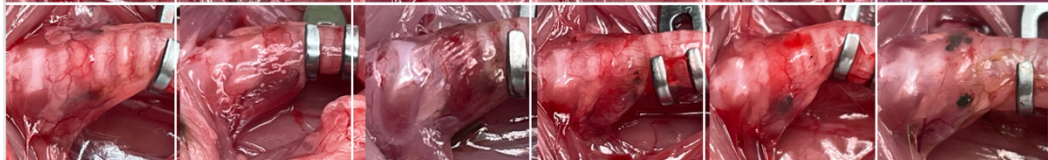

Supplement: pgac087_Supplemental_Files [file pgac087_supplemental_files.zip › PNASNEXUS-PNASNEXUS-2022-00132-s10.pdf]
